# Hyperinsulinemia enhances interleukin-17-induced inflammation to promote prostate cancer development in obese mice through inhibiting glycogen synthase kinase 3-mediated phosphorylation and degradation of interleukin-17 receptor

**DOI:** 10.18632/oncotarget.7296

**Published:** 2016-02-10

**Authors:** Sen Liu, Qiuyang Zhang, Chong Chen, Dongxia Ge, Yine Qu, Rongyi Chen, Yi-Ming Fan, Nan Li, Wendell W. Tang, Wensheng Zhang, Kun Zhang, Alun R. Wang, Brian G. Rowan, Steven M. Hill, Oliver Sartor, Asim B. Abdel, Leann Myers, Qishan Lin, Zongbing You

**Affiliations:** ^1^ Department of Structural and Cellular Biology, Tulane University, New Orleans, LA 70112, USA; ^2^ Department of Dermatology, Affiliated Hospital of Guangdong Medical College, Zhanjiang, Guangdong 524001, China; ^3^ Department of Pathology, Ochsner Clinic Foundation, New Orleans, LA 70130, USA; ^4^ Department of Computer Science and Biostatistics Facility of RCMI Cancer Research Center, Xavier University of Louisiana, New Orleans, LA 70125, USA; ^5^ Department of Pathology and Laboratory Medicine, Tulane University, New Orleans, LA 70112, USA; ^6^ Tulane Cancer Center, Louisiana Cancer Research Consortium, Tulane University, New Orleans, LA 70112, USA; ^7^ Tulane Center for Stem Cell Research and Regenerative Medicine, Tulane University, New Orleans, LA 70112, USA; ^8^ Department of Urology, Tulane University, New Orleans, LA 70112, USA; ^9^ Department of Medicine, Tulane University, New Orleans, LA 70112, USA; ^10^ Department of Biostatistics and Bioinformatics, Tulane University, New Orleans, LA 70112, USA; ^11^ Proteomics/Mass Spectrometry Facility, University at Albany, Rensselaer, NY 12144, USA; ^12^ Department of Orthopaedic Surgery and Tulane Center for Aging, Tulane University, New Orleans, LA 70112, USA

**Keywords:** hyperinsulinemia, obesity, prostate cancer, interleukin-17, GSK3

## Abstract

Interleukin-17 (IL-17) plays important roles in inflammation, autoimmune diseases, and some cancers. Obese people are in a chronic inflammatory state with increased serum levels of IL-17, insulin, and insulin-like growth factor 1 (IGF1). How these factors contribute to the chronic inflammatory status that promotes development of aggressive prostate cancer in obese men is largely unknown. We found that, in obese mice, hyperinsulinemia enhanced IL-17-induced expression of downstream proinflammatory genes with increased levels of IL-17 receptor A (IL-17RA), resulting in development of more invasive prostate cancer. Glycogen synthase kinase 3 (GSK3) constitutively bound to and phosphorylated IL-17RA at T780, leading to ubiquitination and proteasome-mediated degradation of IL-17RA, thus inhibiting IL-17-mediated inflammation. IL-17RA phosphorylation was reduced, while the IL-17RA levels were increased in the proliferative human prostate cancer cells compared to the normal cells. Insulin and IGF1 enhanced IL-17-induced inflammatory responses through suppressing GSK3, which was shown in the cultured cell lines *in vitro* and obese mouse models of prostate cancer *in vivo*. These findings reveal a mechanism underlying the intensified inflammation in obesity and obesity-associated development of aggressive prostate cancer, suggesting that targeting GSK3 may be a potential therapeutic approach to suppress IL-17-mediated inflammation in the prevention and treatment of prostate cancer, particularly in obese men.

## INTRODUCTION

Interleukin-17 (IL-17) family of cytokines includes IL-17A, IL-17B, IL-17C, IL-17D, IL-17E, and IL-17F [1]. There are five receptors, namely, IL-17 receptor A (IL-17RA), IL-17RB, IL-17RC, IL-17RD, and IL-17RE. IL-17RA forms functional heterodimers with IL-17RB (for cytokine IL-17E), IL-17RC (for cytokines IL-17A, IL-17F, and IL-17A/IL-17F heterodimer), IL-17RD (for an unknown ligand), or IL-17RE (for cytokine IL-17C) [2]. IL-17B and IL-17E (also known as IL-25) both bind to IL-17RB [3], and their competition for IL-17RB binding leads to functional antagonism between IL-17B and IL-17E [4]. IL-17A and IL-17F are secreted by T helper 17 (T_H_17) cells, γδ T cells, natural killer cells, and other immune cells [5]. IL-17A and IL-17F act on IL-17RA/IL-17RC receptor complex to recruit nuclear factor-κB (NF-κB) activator 1 (Act1) through SEFIR (similar expression to fibroblast growth factor genes, IL-17 receptors and Toll–IL-1R) domains that exist in IL-17RA, IL-17RC, and Act1 proteins. Act1 is an E3 ubiquitin ligase that activates tumor necrosis factor receptor-associated factor 6 (TRAF6) through lysine-63-linked ubiquitination [6]. The polyubiquitinated TRAF6 then activates transforming growth factor –β-activated kinase 1 (TAK1) and subsequently IκB kinase (IKK) complex, resulting in activation of NF-κB pathway that initiates transcription of a variety of cytokines, chemokines and growth factors, such as C-X-C motif ligand 1 (*CXCL1*), C-C motif ligand 20 (*CCL20*), *IL-1β*, and *IL-6* [7].

The IL-17 cytokine family, particularly IL-17A, plays important roles in a variety of autoimmune diseases, such as rheumatoid arthritis, psoriasis, multiple sclerosis, inflammatory bowel diseases, and systemic lupus erythematosus, and in host defense against bacterial, fungal, and parasitic as well as viral infections [7]. IL-17A promotes organ transplant rejection [8, 9]. IL-17A also promotes development of colon cancer [10–13], skin cancer [14, 15], breast cancer [16], prostate cancer [17, 18], lung cancer [19, 20], and pancreas cancer [21]. IL-17A may be involved with type 2 diabetes [22] and type 1 diabetes [23]. IL-17A may also promote artherosclerosis [24–26]. Many of the aforementioned conditions are commonly seen in obesity [27, 28]. Obesity is a chronic inflammatory state with increased serum levels of inflammatory cytokines TNFα and IL-6 [29]. Obese people have elevated serum levels of IL-17, insulin, and insulin-like growth factor 1 (IGF1) [29–31]. Insulin and IGF1 activate Akt to inhibit glycogen synthase kinase 3 (GSK3) activities [32], thus enhancing IL-17-induced expression of *Cxcl1*, *Ccl20*, and *Il-6* [33, 34]. GSK3 inhibitors also enhance IL-17-induced gene expression [33, 35], whereas GSK3β overexpression or Akt inhibitors repress IL-17-induced gene expression [33–35]. These findings suggest that GSK3 mediates the crosstalk between IL-17 and insulin/IGF1 signaling pathways. Indeed, *Gsk3α*- or *Gsk3β*-knockout abolishes the synergy between IL-17 and insulin/IGF1 signaling pathways [33, 34]. However, the molecular mechanisms underlying how GSK3 mediates the crosstalk are largely unknown. Here we demonstrate that GSK3 constitutively binds to and phosphorylates IL-17RA at T780, leading to ubiquitination and proteasome-mediated degradation of IL-17RA, thus inhibiting IL-17-mediated inflammation. Insulin and IGF1 enhance IL-17-induced inflammatory responses through suppressing GSK3, leading to enhanced prostate cancer formation in obese mice.

## RESULTS

### Obese mice develop more invasive prostate cancers than lean mice

**Figure 1:**
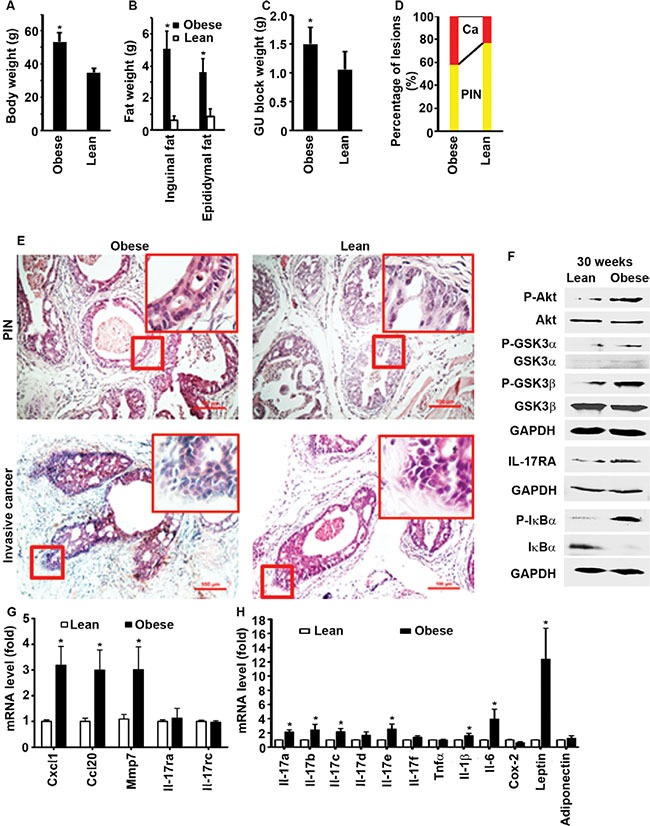
High-fat diet-induced obesity promotes prostate cancer formation in Pten conditional knockout mouse prostates (**A**) Mouse body weight at age of 30 weeks. *P < 0.001 (Student's *t* test). (**B**) The tissue weight of inguinal fat and epididymal fat. *P < 0.001 (Student's *t* test). (**C**) The genitourinary (GU) block weight. *P < 0.01 (Student's *t* test). (**D**) Percentages of PIN and invasive cancer in dorsal, lateral, and ventral prostatic lobes. *P < 0.001 (Kruskal–Wallis test). (**E**) Representatives of H & E-stained dorsal prostatic lobes showing mouse prostatic intraepithelial neoplasia (PIN) and invasive (or microinvasive) adenocarcinomas; the red windows highlight the selected regions at x 400 magnification; scale bar, 100 μm. (**F**) Western blot analysis of the proteins isolated from the representative prostate tissues of obese and lean mice at age of 30 weeks. Anti-P-GSK3α antibodies recognize Ser21 phosphorylation site and anti-P-GSK3β antibodies recognize Ser9 phosphorylation site. (G,H) qRT-PCR analysis of mRNA expression in lean and obese mouse prostates. *P < 0.05 (one-way ANOVA). Data represent mean ± standard deviation of 10 mice per group (*n* = 10).

Obese men are at increased risk of developing aggressive prostate cancer and dying of prostate cancer [36]. To simulate human prostate cancer development, we used a previously established mouse model that was crossbred between male mice with probasin promoter-driven Cre recombinase and female mice with floxed phosphatase and tensin homolog (*Pten*), thus *Pten* was conditionally knocked out in the mouse prostate, leading to prostate adenocarcinoma formation [17, 18, 37]. Starting at 3 weeks of age after weaning, male mice fed with standard chow diet (13.2% calories by fat) for 27 weeks (i.e., at 30 weeks of age) had an average body weight of 34.9 ± 2.5 g (*n* = 10, named as lean mice). In contrast, male mice fed with high-fat diet (60% calories by fat) for 27 weeks (i.e., at 30 weeks of age) had an average body weight of 53.2 ± 5.7 g (*n* = 10, named as obese mice), which was approximately 52% more than lean mice (Figure 1A, *P* < 0.001). Obese mice had significantly more inguinal and epididymal fat tissues than lean mice (Figure 1B, *P* < 0.001). The genitourinary (GU) block weight (a surrogate measurement of prostate tumor weight) was significantly heavier in obese mice than lean mice (Figure 1C, *P* < 0.01). Invasive (or microinvasive) adenocarcinomas were found in approximately 42% of the prostatic glands in obese mice, but only in 23% of those in lean mice (Figure 1D–1E, *P* < 0.001). These findings suggest that obese mice developed more invasive prostate cancers than lean mice, which is consistent with the published reports using Hi-Myc mouse model [38, 39]. Plasma levels of leptin (obese mice = 0.9 ± 0.4 versus lean mice = 0.2 ± 0.2 ng/ml, *n* = 10, *P* < 0.001) and insulin (obese mice = 300.2 ± 150.4 versus lean mice = 147.5 ± 25.5 μIU/ml, *n* = 10, *P* = 0.015) were significantly higher in obese mice than lean mice. Plasma IL-17A (obese mice = 248.3 ± 98.8 versus lean mice = 350.3 ± 148.7 pg/ml, *n* = 10, *P* = 0.088) and IGF1 (obese mice = 8.6 ± 1.7 versus lean mice = 6.9 ± 2.2 ng/ml, *n* = 10, *P* = 0.071) levels were not significantly different between obese and lean mice. Obese mouse prostate tissues had increased levels of P-Akt, P-GSK3α/β, and *Il-17*-downstream genes such as *Cxcl1*, *Ccl20*, matrix metalloproteinase 7 (*Mmp7*), *Il-1β*, and *Il-6* (Figure 1F–1H). *Il-17ra* and *Il-17rc* mRNA levels were not different between obese and lean mouse prostates (Figure 1G), but IL-17RA protein levels were higher in obese mouse prostates than lean mouse prostates (Figure 1F). P-IκBα levels were increased while IκBα levels were decreased in obese mouse prostates (Figure 1F), indicating activation of NF-κB in obese mouse prostates. These findings suggest that increased IL-17RA levels in obese mouse prostates may be responsible for the enhanced expression of *Il-17*-downstream genes that promote formation of invasive prostate cancer in obese mice. IL-17RA level is also increased in human prostate cancer [40]. It was not known how IL-17RA protein level was increased in the absence of an increase at the *Il-17ra* mRNA level, which prompted us to conduct further mechanistic studies as described below.

### GSK3β constitutively binds to and phosphorylates IL-17RA

**Figure 2:**
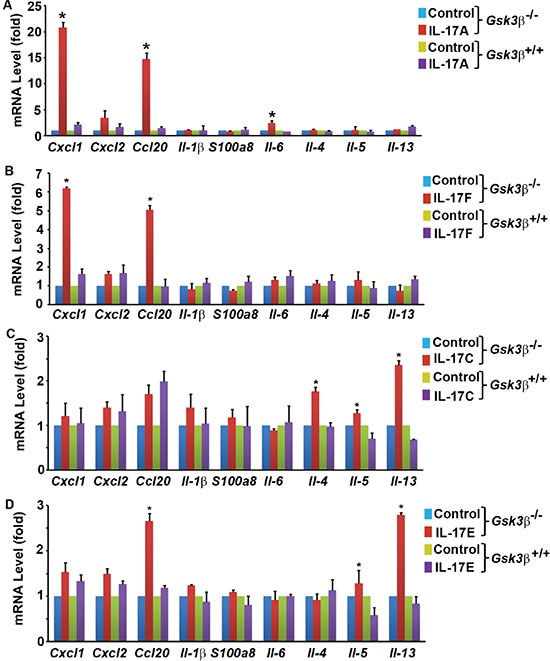
Induction of gene expression in *Gsk3β^+/+^* (wild-type) and *Gsk3β^−/−^* (knockout) mouse embryonic fibroblasts (MEFs) by IL-17 family cytokines (**A**–**D**) MEFs were treated with 20 ng/ml mouse recombinant IL-17A (A), IL-17F (B), IL-17C (C), and IL-17E (D) for 2 h. Gene expression was determined using qRT-PCR analysis. The levels of the control group (treated with phosphate-buffered saline) were taken as the basal levels. Data represent the mean ± standard deviation of three independent experiments (*n* = 3); *P < 0.05 (Student's *t* test).

**Figure 3:**
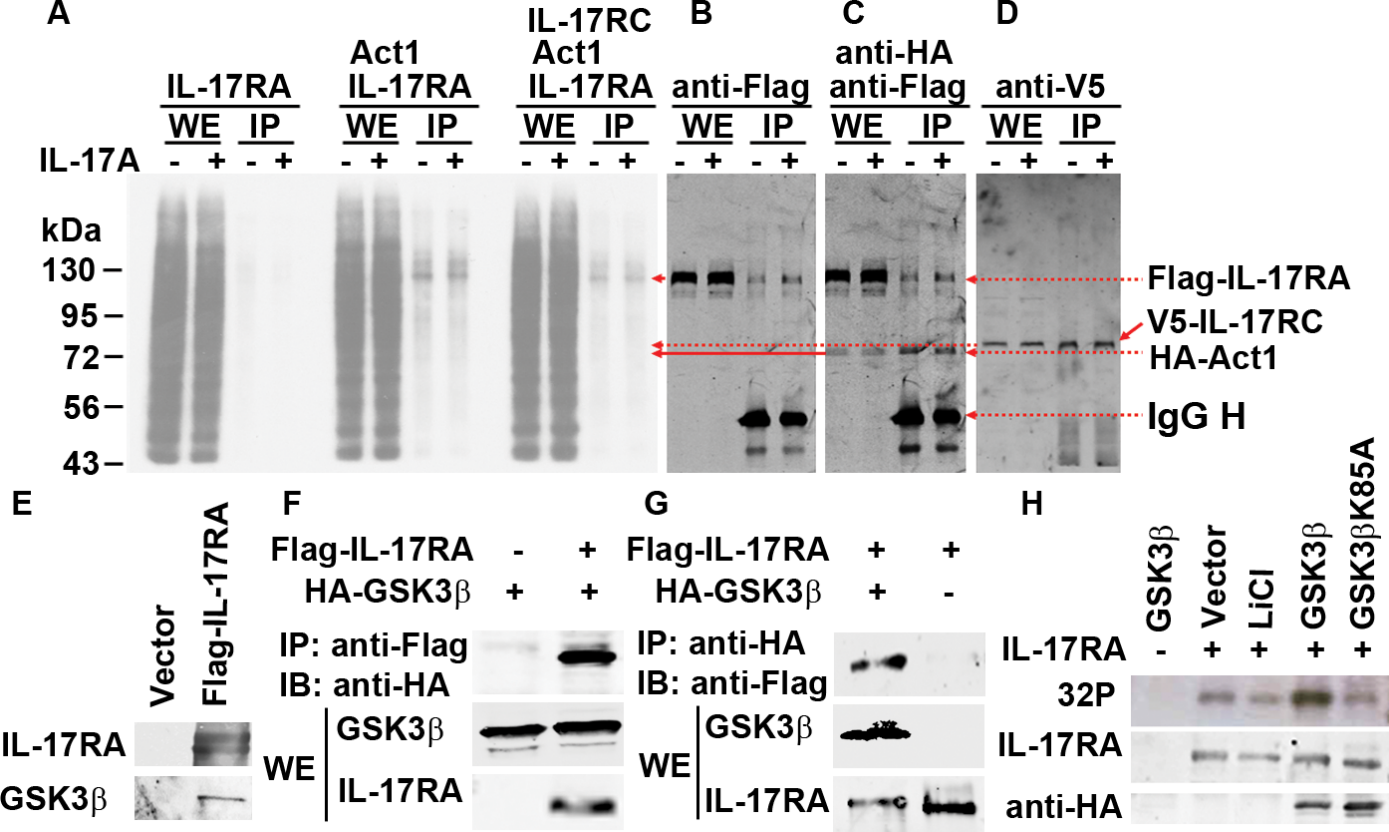
GSK3β binds to and phosphorylates IL-17RA (**A**–**D**) 293 cells were co-transfected with Flag-IL-17RA, V5-IL-17RC, and HA-Act1, labeled with ^32^P orthophosphate, and treated with 20 ng/ml IL-17A for 20 min; IP with anti-HA; autoradiography (A) followed by IB (B-D). (**E**) Anti-Flag IP followed by IB. (**F**–**G**) Co-IP of Flag-IL-17RA and HA-GSK3β. (**H**) 293 cells were co-transfected with Flag-IL-17RA, WT HA-GSK3β or kinase-dead mutant HA-GSK3βK85A, labeled with ^32^P orthophosphate, or treated with 20 mM LiCl for 2 h; autoradiography (^32^P) followed by IB. WE, whole cell extract.

We and other investigators have shown that GSK3 represses IL-17A-induced gene expression [33–35]. We previously demonstrated that insulin and IGF1 activate Akt to inhibit GSK3 activities, thus enhancing IL-17A-induced expression of *Cxcl1*, *Ccl20*, and *Il-6* [33, 34]. We found that in *Gsk3α*- or *Gsk3β*-knockout mouse embryonic fibroblasts (MEFs), the synergy between IL-17A and insulin/IGF1 is abolished, but both *Gsk3α*- and *Gsk3β*-knockout enhance IL-17A-induced gene expression [33, 34]. In our further analysis, we found that *Gsk3β*-knockout enhanced not only gene expression induced by IL-17A and IL-17F (i.e., *Cxcl1* and *Ccl20*), but also that induced by IL-17C (i.e., *Il-4* and *Il-13*) and IL-17E (i.e., *Ccl20*, *Il-5*, and *Il-13*) (Figure 2A–2D), suggesting that GSK3β restricts the signaling of the whole IL-17 cytokine family at an apical level and likely at IL-17RA receptor as it binds to IL-17A/IL-17F, IL-17C, and IL-17E [2]. However, IL-17RA is not known as a GSK3 substrate. Act1, an IL-17RA and IL-17RC binding protein, is phosphorylated [41, 42]. To determine the candidate(s) of GSK3 substrate, we transfected human embryonic kidney 293 cells with Flag-tagged IL-17RA, V5-tagged IL-17RC, and/or HA-tagged Act1, and labeled the cells with [^32^P] orthophosphate. Immunoprecipitation (IP) was performed using anti-HA antibody to pull down Act1 and its binding partners IL-17RA and IL-17RC. After autoradiography to show ^32^P labeling, the same blot was used to detect Flag-IL-17RA, V5-IL-17RC, and HA-Act1 by immunoblotting (IB). To our surprise, we found that only Flag-IL-17RA was strongly labeled with ^32^P (Figure 3A–3D and Supplementary Figure S1A), which was reduced by calf-intestinal alkaline phosphatase (CIP) treatment (Supplementary Figure S1B). An algorithm analysis predicted that IL-17RA contained many potential phosphorylation sites including multiple GSK3 consensus sites (Supplementary Table S1). In 293 cells transfected with Flag-IL-17RA, anti-Flag IP pulled down GSK3β (Figure 3E), and further Co-IP assays confirmed binding of GSK3β to IL-17RA (Figure 3F–3G). To check if GSK3 was functional in IL-17RA phosphorylation, 293 cells were co-transfected with Flag-IL-17RA, HA-GSK3β, or HA-GSK3βK85A (a kinase-dead mutant of GSK3β), and labeled with [^32^P] orthophosphate, with or without LiCl (a GSK3 inhibitor) treatment. We found that LiCl treatment reduced ^32^P labeling of Flag-IL-17RA, but overexpression of wild-type (WT) HA-GSK3β enhanced ^32^P labeling of Flag-IL-17RA, while overexpression of kinase-dead HA-GSK3βK85A did not show any effects (Figure 3H). Together, these data demonstrate that GSK3β constitutively binds to and phosphorylates IL-17RA.

### T780 is one of the IL-17RA phosphorylation sites

**Figure 4:**
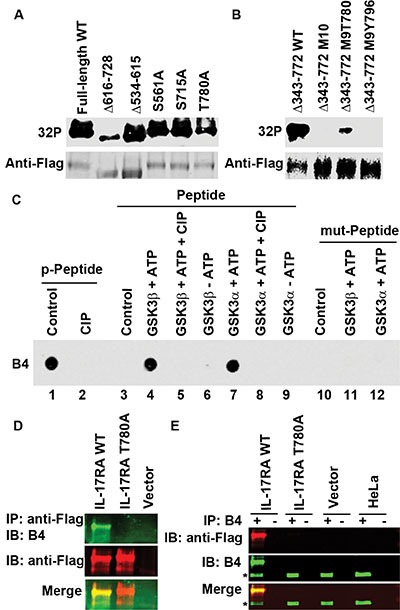
GSK3β phosphorylates IL-17RA at T780 (**A**–**B**) 293 cells were transfected with Flag-IL-17RA and its mutants and labeled with ^32^P orthophosphate; autoradiography (^32^P) followed by IB. (**C**) IL-17RA peptide phosphorylated at T780 (p-Peptide) was treated with CIP, while IL-17RA peptide with wild-type T780 (Peptide) or with mutant T780A (mut-Peptide) were treated with recombinant GSK3, followed with dot blot analysis using B4 antibodies. (**D**–**E**) Flag-IL-17RA, Flag-IL-17RAT780A mutant, or empty vector was transfected into 293 cells; HeLa cells were not transfected; IP with anti-Flag, B4 (+), or control IgG (−), followed by IB; *indicates endogenous P-IL-17RA.

To identify the phosphorylation sites of IL-17RA, we purified Flag-IL-17RA protein expressed in 293 cells. Mass spectrometry analysis of the purified protein identified 8 phosphorylation sites including S554, S629, S708, S726, T780, S792, S798, and S801, all of which are conserved between human and mouse except S708 and S726 (Supplementary Figure S2). To confirm these sites, we made IL-17RA mutants. Deletion of residues from 616 to 728 (Δ616-728) or T780A mutation reduced ^32^P labeling (Figure 4A), suggesting that there are multiple phosphorylation sites and T780 is one of them. To pinpoint the sites, we deleted the proximal intracellular 430 amino acids (Δ343-772) and mutated all 10 potential phosphorylation sites within the remaining C-terminal 94 amino acids (Δ343-772 M10) (Supplementary Figure S1C). Δ343-772 WT protein was labeled with ^32^P that was reduced by CIP (Supplementary Figure S1D). Δ343-772 M10 protein was not labeled with ^32^P (Figure 4B); however, when T780 was reversed to WT in the protein (Δ343-772 M9T780), ^32^P labeling re-appeared (Figure 4B), confirming that T780 is one of the phosphorylation sites.

### T780 is phosphorylated by GSK3 and is detectable with anti-P-IL-17RA (T780) antibodies

Next, we generated rabbit polyclonal antibodies against a phosphopeptide (C-LTDPH_P_TPYEEEQ) in four rabbits, which were named as B1, B2, B3, and B4 antibodies (Supplementary Figure S1E). All of them recognized the phosphopeptide, but not the unphosphorylated peptide or CIP-treated phosphopeptide (Supplementary Figure S1F–S1G). WT peptide, but not the T780A mutant peptide, was phosphorylated by recombinant GSK3α and GSK3β proteins in *in vitro* kinase assays (Figure 4C). B4 could detect WT Flag-IL-17RA, but not any mutants containing the T780A mutation (Figure 4D and Supplementary Figure S1H). B4 also detected endogenous P-IL-17RA in 293 and HeLa cells (Supplementary Figure S1I). B4 IP pulled down exogenous and endogenous P-IL-17RA, but not T780A-mutant IL-17RA (Figure 4E). These data demonstrate that T780 is phosphorylated by GSK3, which is detectable with anti-P-IL-17RA (T780) antibodies.

### Insulin reduces the levels of P-IL-17RA through suppressing GSK3

**Figure 5:**
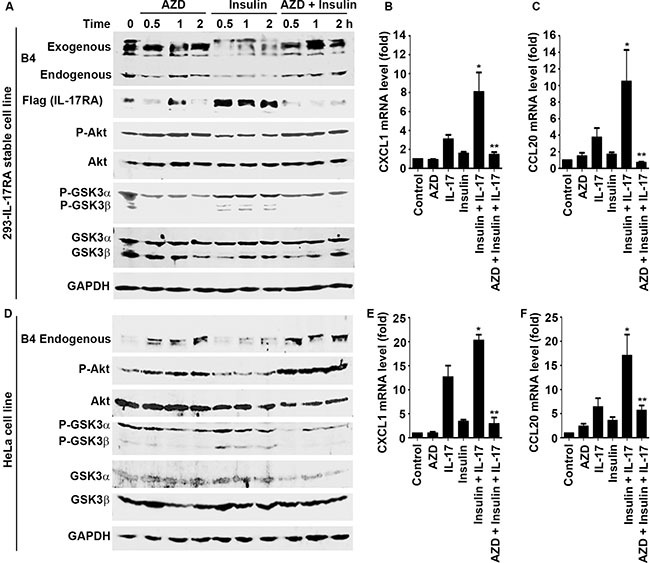
AZD5363 antagonizes insulin-induced enhancement of IL-17 responses through restoring IL-17RA phosphorylation (**A**) and (**D**) 239 cells stably expressing Flag-IL-17RA (293-IL-17RA cell line) and HeLa cells were treated with 2 μM AZD5363 (a pan-Akt inhibitor) and/or 50 ng/ml insulin for the indicated time periods; Western blot analysis was performed for the indicated proteins; exogenous, IL-17RA transfected into 293 cells; endogenous, endogenous IL-17RA expressed in 293 and HeLa cells. (**B**) and (**C**) 293-IL-17RA cells were treated with 20 ng/ml IL-17A, with or without 2 μM AZD5363 and/or 50 ng/ml insulin for 2 h; IL-17-downstream gene expression was determined using qRT-PCR analysis; *P < 0.05 compared to each single treatment group; **P < 0.05 compared to the combined insulin and IL-17 treatment group (one-way ANOVA). (**E**) and (**F**) HeLa cells were treated with 20 ng/ml IL-17A, with or without 2 μM AZD5363 and/or 50 ng/ml insulin for 2 h; IL-17-downstream gene expression was determined using qRT-PCR analysis; *P < 0.05 compared to each single treatment group; **P < 0.05 compared to the combined insulin and IL-17 treatment group (one-way ANOVA). Data represent the mean ± standard deviation of three independent experiments (*n* = 3).

AZD5363 (a pan-Akt inhibitor) can inhibit Akt, thus increasing GSK3 activity, whereas insulin can activate Akt to inhibit GSK3 activity [34]. AZD5363 increased the levels of exogenous P-IL-17RA in 293 cells and endogenous P-IL-17RA in HeLa cells (Figure 5A and 5D). In contrast, insulin treatment reduced the levels of exogenous and endogenous P-IL-17RA, while increasing the levels of total exogenous IL-17RA (Figure 5A). Consequently, a combination of insulin and IL-17 treatment significantly enhanced IL-17-downstream gene expression, whereas AZD5363 inhibited this synergy in both 293-IL-17RA stable cells (Figure 5B and 5C) and HeLa cells (Figure 5E and 5F). These data suggest that insulin reduces the levels of P-IL-17RA through suppressing GSK3, leading to increased levels of total IL-17RA and IL-17 responsiveness.

### Phosphorylation of IL-17RA at T780 leads to its ubiquitination and proteasome-mediated degradation

**Figure 6:**
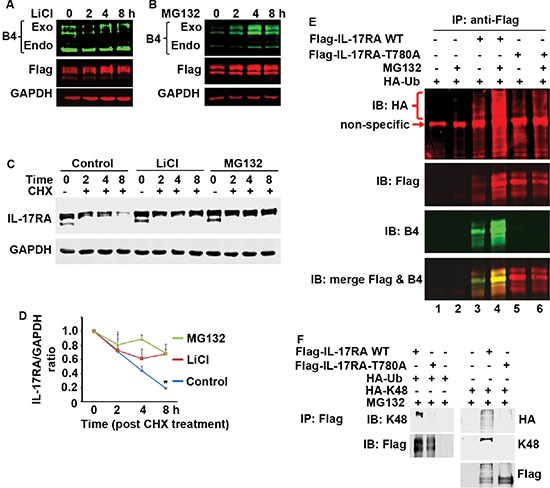
Phosphorylated IL-17RA is ubiquitinated and degraded by proteasome-mediated mechanism (**A**–**D**) 293 cells stably expressing Flag-IL-17RA were treated with 20 mM LiCl or 10 μM MG132, and/or 50 μg/ml CHX; P-IL-17RA was detected using B4 and total exogenous IL-17RA was detected using anti-Flag; the levels of total exogenous IL-17RA (C) were normalized by the levels of GAPDH (D); exo, exogenously transfected IL-17RA; endo, endogenously expressed IL-17RA; *P < 0.05 compared to LiCl and MG132 treated groups (Student's *t* test). (**E**) Flag-IL-17RA and Flag-IL-17RA-T780A mutant were co-transfected with HA-tagged ubiquitin (HA-Ub), with or without 10 μM MG132 treatment; non-specific, an unknown band detected by anti-HA antibodies, which was not IL-17RA or IgG heavy chain (see Supplementary Figure S3C). (**F**) Flag-IL-17RA and Flag-IL-17RA-T780A mutant were co-transfected with HA-tagged ubiquitin (HA-Ub) or K48-only ubiquitin (HA-K48), with 10 μM MG132 treatment, followed by IP and IB.

LiCl, a GSK3 inhibitor, reduced the levels of exogenous and endogenous P-IL-17RA, while increased the total exogenous IL-17RA (Figure 6A). MG132, a proteasome inhibitor, increased the levels of both phosphorylated and total exogenous IL-17RA (Figure 6B). When treated with cycloheximide (CHX, a protein synthesis inhibitor), the total exogenous IL-17RA level decreased rapidly, while LiCl or MG132 inhibited the decrease (Figure 6C and 6D). These data suggest that phosphorylated IL-17RA undergoes proteasome-mediated degradation. Further, degradation of Δ343-772 M9T780 protein was faster than Δ343-772 M10 protein (Supplementary Figure S3A–S3B). WT Flag-IL-17RA was ubiquitinated, the level of which was increased in the presence of MG132 (Figure 6E, lanes 3 versus 4); however, IL-17RA T780A mutant showed minimal ubiquitination even in the presence of MG132 (Figure 6E, lanes 5 versus 6). Further, WT IL-17RA, but not T780A mutant, was ubiquitinated with K48-linked ubiquitin (Figure 6F) and K63-linked ubiquitin (Supplementary Figure S3D). These data demonstrate that phosphorylation of IL-17RA at T780 leads to its ubiquitination and proteasome-mediated degradation, resulting in inhibition of IL-17-mediated inflammatory responses.

### Phosphorylation of IL-17RA is reduced in proliferative prostate cancer cells and keratinocytes

**Figure 7:**
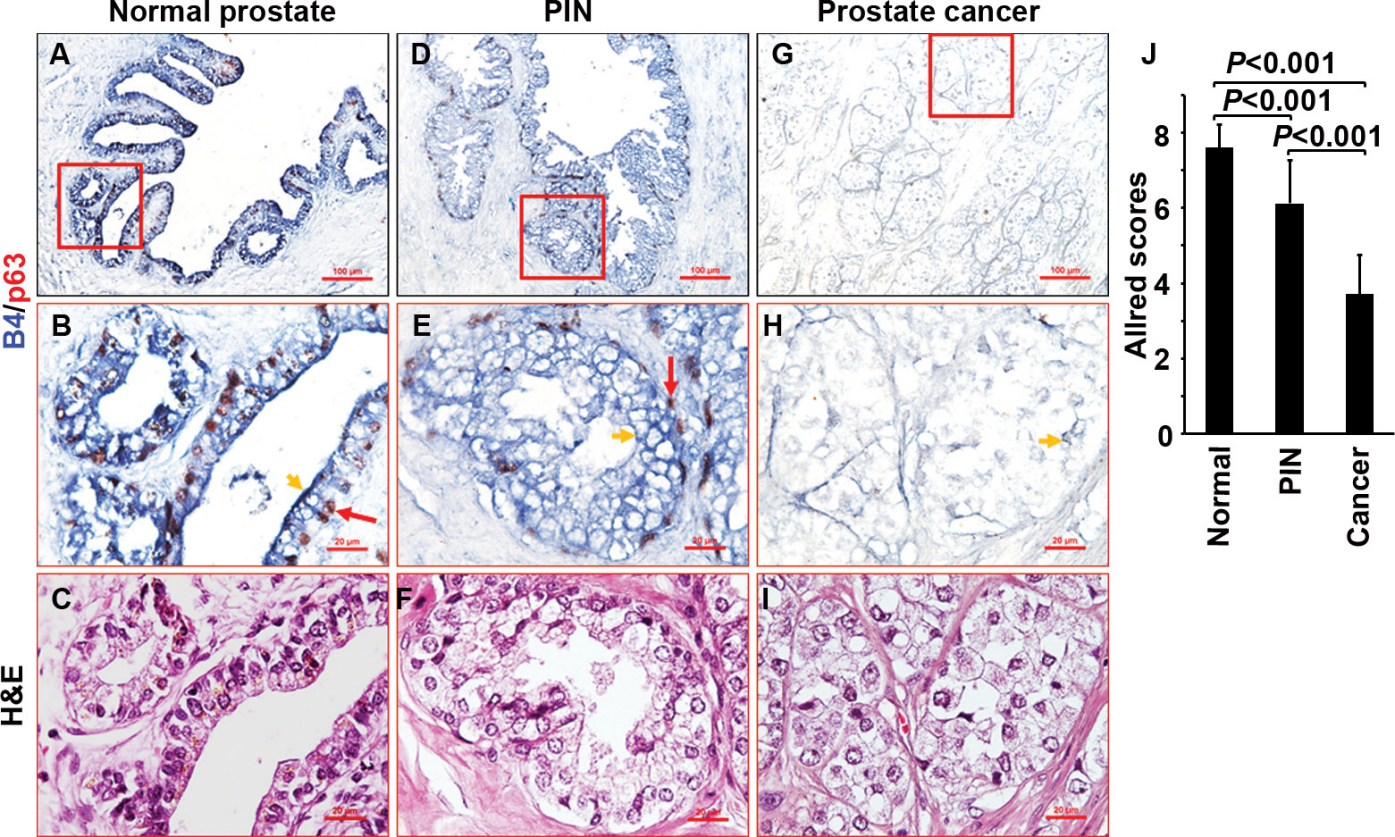
Phosphorylation of IL-17RA is detected in human prostate tissues (**A**–**I**) Human normal prostate, PIN, and prostate cancer tissues were double stained for P-IL-17RA using B4 (arrowheads) and for basal cells using anti-p63 (arrows). (**J**) Quantification of P-IL-17RA staining.

IL-17-mediated inflammation plays important roles in human and mouse prostate cancers [17, 18, 40, 43, 44]. Human normal prostatic epithelium was stained highly positive for P-IL-17RA (Figure 7A–7C). Human prostatic intraepithelial neoplasia (PIN) was stained moderately positive for P-IL-17RA (Figure 7D–7F). In contrast, human prostate cancer was stained weakly positive for P-IL-17RA (Figure 7G–7I and Supplementary Figure S4A–S4H). On the same tissue section containing both malignant and normal glands (identified by histology and presence of p63-positive basal cells), the malignant glands were stained weakly for P-IL-17RA but strongly for IL-17RA, whereas the normal glands were stained strongly for P-IL-17RA but weakly for IL-17RA (Supplementary Figure S4I–S4Q). Using the Allred scoring system [45], P-IL-17RA staining was significantly higher in human normal prostate and PIN than prostate cancer (Figure 7J). IL-17 also stimulates rapid proliferation of keratinocytes in psoriasis [46]. In both human normal and psoriatic epidermis, the differentiated suprabasal keratinocytes were stained strongly positive for P-IL-17RA, whereas the proliferating basal keratinocytes were stained negative for P-IL-17RA (Supplementary Figure S5A–S5J). B4/Ki-67 double staining showed that Ki-67-positive keratinocytes were not stained by B4, while B4-positive keratinocytes were not stained by Ki-67 (Supplementary Figure S5D and S5J). In psoriatic epidermis, Ki-67-positive keratinocytes were stained negative for P-IL-17RA but positive for IL-17RA (Supplementary Figure S5K–S5P). These data demonstrate that the levels of P-IL-17RA are reduced while the levels of IL-17RA are increased in proliferative prostate cancer cells and keratinocytes, which may enhance IL-17 responsiveness.

### Phosphorylation by GSK3 is conserved in human and mouse IL-17RA

Mouse P-IL-17RA levels were not assessable due to lack of anti-mouse P-IL-17RA antibodies. However, mouse peptide containing T779 (corresponding to human T780) was phosphorylated by recombinant GSK3α and GSK3β (Supplementary Figure S6A–S6B). Purified full-length mouse IL-17RA was phosphorylated by recombinant GSK3α and GSK3β, which was very weakly cross-reactive with B4 antibodies (Supplementary Figure S6C), due possibly to the similarity between mouse and human epitopes and the high levels of phosphorylated mouse IL-17RA in the *in vitro* assays. However, B4 antibodies could not detect the endogenous levels of mouse P-IL-17RA (Supplementary Figure S6C, the control lane).

## DISCUSSION

The present study shows that GSK3 binds to and phosphorylates IL-17RA at T780, leading to K48-linked ubiquitination and proteasome-mediated degradation of IL-17RA. Thus, GSK3 represses IL-17 signaling through reducing IL-17RA protein levels (Supplementary Figure S6D). This mechanism explains our previous observation that insulin and IGF1 enhance IL-17-induced gene expression [33, 34], as insulin and IGF1 can activate PI3K/Akt to suppress GSK3 activities (Supplementary Figure S6D). Obese people have elevated serum levels of IL-17, insulin, and IGF1 [29–31], as well as increased serum levels of inflammatory cytokines TNFα and IL-6 that are IL-17-downstream target genes. Our findings suggest that in obesity, insulin and IGF1 signaling pathway cross-talks with IL-17 signaling pathway through suppressing GSK3 activities, thus enhancing IL-17-mediated inflammation and contributing to the chronic inflammatory status commonly seen in obesity. In our obese animal model, increased insulin levels activate Akt to increase inhibitory phosphorylation of GSK3α/β, leading to reduced phosphorylation of IL-17RA and hence reduced ubiquitination and degradation of IL-17RA. Subsequently, IL-17RA protein levels are increased to enhance IL-17 responsiveness as evidenced by increased NF-κB activation and enhanced expression of IL-17-downstream genes such as *Cxcl1*, *Ccl20*, *Il-6*, and *Mmp7*. The biological consequence is development of more invasive prostate cancers in obese mice than lean mice, which echoes the observation that obese men are at increased risk of developing aggressive prostate cancer [36].

To our best knowledge, this is the first study showing that IL-17RA is phosphorylated. It appears that there are multiple phosphorylation sites of IL-17RA, though we only focused on T780 residue. We presented several lines of evidence to confirm that T780 is phosphorylated by GSK3: first, ^32^P labeling is reduced with T780A mutant IL-17RA compared to wild-type IL-17RA; second, reversal of Δ343-772 M10 (with all potential phosphorylation sites mutated) to Δ343-772 M9T780 (with only T780 reversed to wild-type) reinstalled ^32^P labeling; third, T780 wild-type peptide, but not T780A mutant peptide, is phosphorylated by GSK3α/β; and fourth, wild-type, but not T780A mutant, IL-17RA is recognized by the antibodies against T780-phosphorylated IL-17RA. Phosphorylation of IL-17RA at T780 is linked to K48-mediated ubiquitination and proteasome-mediated degradation, as we showed that T780A mutant IL-17RA is less ubiquitinated and degraded compared to wild-type IL-17RA. IL-17RA is also ubiquitinated with K63 ubiquitin, and T780A mutant IL-17RA also showed reduced K63-mediated ubiquitination. The biological significance of K63-ubiquitination of IL-17RA is unknown, though K63-ubiquitination of other proteins has been linked to protein functions [6]. It has also been demonstrated that both K48- and K63-ubiquitinations are associated with endocytosis and degradation of some membrane proteins [47, 48].

Phosphorylation of IL-17RA is linked to degradation of IL-17RA. We have demonstrated that total IL-17RA protein levels are increased when IL-17RA phosphorylation is reduced by LiCl and insulin through inhibiting GSK3 activities. Conversely, when IL-17RA phosphorylation is increased by AZD5363 treatment, the total IL-17RA protein level is reduced. Thus, there is an inverse correlation between IL-17RA and P-IL-17RA levels. We observed that this is true in human prostate and skin tissues. The levels of P-IL-17RA are higher in the normal prostatic glands than prostate cancer. In contrast, the levels of IL-17RA are higher in prostate cancer than in the normal prostatic glands, which is consistent to a previous report [40]. We found that obese mouse prostate tumors expressed higher levels of IL-17RA than lean mice (Figure 1F). We also observed that IL-17RA levels were mainly increased in the epithelial tumor cells, though IL-17RA levels in the stromal cells were also increased in obese mice compared to lean mice (IHC data not shown). In human prostate cancer, it has been shown that GSK3α is mainly expressed in low-risk prostate cancers and is associated with hormone-dependent androgen receptor (AR)-mediated gene expression, whereas GSK3β is mainly expressed in high-risk prostate cancers and is associated with hormone-independent AR-mediated gene expression [49, 50]. Knockout either *Gsk3α* or *Gsk3β* can increase the IL-17 responsiveness in mouse embryonic fibroblasts [33, 34]. However, in mouse prostate tumors, GSK3β levels are higher than GSK3α levels (Figure 1F), thus GSK3β may play more important roles than GSK3α. It is intriguing to investigate whether there is any link between IL-17RA and AR in the context of GSK3's regulatory functions in prostate cancer. In normal and psoriatic skin tissues, P-IL-17RA is absent in the proliferative keratinocytes, while IL-17RA is present. The levels of IL-17RA are associated to IL-17 responsiveness as shown in our *in vitro* studies with 293-IL-17RA and HeLa cell lines. Treatment of the cells with insulin increases the levels of IL-17RA and the expression levels of IL-17-induced genes, whereas AZD5363 treatment decreases the levels of IL-17RA and the expression levels of IL-17-induced genes.

In summary, our observation that IL-17RA is phosphorylated by GSK3 reveals a new role of GSK3 in the crosstalk between the metabolic pathway and inflammatory pathway. Targeting GSK3, e.g., using melatonin or AZD5363 [33, 34], represents a potential therapeutic approach to suppress IL-17-mediated inflammation that may be applicable to the prevention and treatment of prostate cancer.

## MATERIALS AND METHODS

### Reagents

The antibodies and peptides used are as follows: rabbit anti-human phospho-IL-17RA (T780) polyclonal antibodies (B1, B2, B3, and B4), phosphorylated antigen peptide (C-LTDPH(pT)PYEEEQ) and non-phosphorylated antigen peptide (C-LTDPHTPYEEEQ) were produced through a contract by AbMART (Shanghai) CO., LTD., Shanghai, China. The same antigen peptides and mutant peptide (C-LTDPH(A)PYEEEQ) were independently synthesized by Peptide 2.0 Inc. (Chantilly, VA, USA), which were used for characterization of the antibodies. Rabbit anti-P-Akt (S473), rabbit anti-Akt, rabbit anti-P-GSK3β (S9), rabbit anti-GSK3β, rabbit anti-P-GSK3α (S21), and rabbit anti-GSK3α antibodies were purchased from Cell Signaling Technology. Mouse anti-GAPDH antibodies were purchased from Millipore Corporation. Mouse anti-Flag antibodies and ANTI-FLAG^®^ M2 Affinity Gel were purchased from Sigma Aldrich. Mouse anti-HA antibodies were purchased from Covance. Rabbit and mouse anti-Ki-67 and anti-IL-17RA (H-168) antibodies were purchased from Santa Cruz Biotechnology. Mouse anti-p63 antibodies were purchased from Biocare Medical.

### Plasmids and construction of IL-17RA mutants

The plasmids used were as follows: pcDNA3.1 expressing human IL-17RA with Flag tag and pcDNA3.1 expressing human Act1 with HA tag were described previously [51] and were kindly provided by Dr. Xiaoxia Li at Cleveland Clinic Lerner Research Institute. Constructs with point mutations and truncations were created by overlapping extension PCR, using the Flag-IL-17RA plasmid as template. The sense strand primers are as follows: IL-17RA-T780A forward: 5′gtcctcacagacccacacgcgccctacgaggaggagcagcgg 3′; IL-17RAΔ781-820T780A forward:5′ gtcctcacagacccac acgcgccagggaagccggccctgcca 3′; IL-17RA-796A+798A +800A+801A forward: 5′ cagtctgaccagggcgccatcgccagg gccgccccgcagccccccgag 3′; IL-17RA-Y796A forward: 5′ cagtctgaccagggcgccatctccaggagctcc 3′; IL-17RA-T780A +Y782A+S789A+S 792A forward: 5′ gtcctcacagacccaca cgcccccgccgaggaggagcagcggcaggccgtgcaggccgaccaggg ctacatc 3′; IL-17RA-S 792A+ Y796A+ S800A forward: 5′cggcagtcagtgcaggccgaccagggcgccatctccagggcctccccgca gcccccc 3′; IL17RA-S861A+S865A forward: 5′ gcgcc cctggtgcgcgagcctggcgcccaggcctgcctggccatagacccgc 3′; IL-17RAΔ534-615 forward:5′ gacaggttcgaggaggtgtacggca tcgtgaagcgggcgccc 3′; IL-17RAΔ616-728 forward: 5′ ccactgctgcctccgggaaccggcagcagcacccccatggcg 3′; IL-17 RAΔ729-780 forward: 5′ gaccccgaggactcgccccttgtcctcaca gacccacacacg 3′; IL-17RAΔ343-772 forward: 5′ ctgctcatcg tctgcatggtcctcacagacccacac 3′; IL-17RA-S561A forward: 5′ gacaactacctgcgggccccgggcggcaggcag 3′; IL-17 RA-S715A forward: 5′ ggcgctgggcgaaatgccgtcct cttcctcccc 3′; IL-17RA-S554A+Y558A+S561A forward: 5′ cgcgtaggggagctggcgggggacaacgccctgcgggccccgggcggca ggcag 3′; IL-17RA-Y591A-S592A-S600 forward: 5′ gaatgtgagaacctcgccgccgcagatgaccaggatgccccggccctggacga agaggtg 3′. The antisense primers were their complementary sequences. All of the primers were synthesized by Eurofins Genomics. Mutagenic reactions were performed in 20 μl of PCR mix containing 20–40 ng of template DNA, 100 nM primers and 2.5 U Pfx DNA polymerase (Invitrogen). The PCR conditions were: an initial denaturation at 94°C for 5 min, followed by 18 cycles of 94°C for 1 min, 60°C for 1 min, and 68°C for 12 min as well as a final extension at 68°C for 20 minutes. 1 μl (20U) of DpnI enzyme (New England Biolab) was added to the PCR products at 37°C for 2 h. After DpnI digestion, 5 μl PCR products were transformed into 50 μl of DH5α E. coli competent cells (Invitrogen). Then, 250 μl of LB medium (Invitrogen) were added for recovery at 37°C for 1 h. The transformed bacteria were plated on LB agar (Invitrogen) plates with ampicillin antibiotics. After overnight culture at 37°C, two or three colonies were cultured for plasmid DNA isolation, using Wizard Plus SV minipreps purification system (Promega). All constructs were confirmed by DNA sequencing (Genewiz, Inc.).

### Cell culture, transfection and establishment of stable cell lines

Human cervical cancer HeLa cell line and human embryonic kidney 293 cell line were obtained from the American Type Culture Collection, Manassas, VA, and were free of mycoplasma contamination. The cells were maintained in Dulbecco's Modified Eagles Medium (DMEM, Mediatech, Inc.) containing 10% fetal bovine serum (FBS, Mediatech, Inc.) and 100 IU/ml penicillin/streptomycin in a 37°C, 5% CO_2_ humidified incubator. Transient overexpression transfections were performed using Lipofectamine 2000 (Invitrogen) according to the manufacturer's instructions. 293 cells were used to establish cell lines that stably expressed wild-type Flag-IL-17RA, Flag-IL-17RA T780A mutant, and other mutants. Twenty-four h prior to transfection, 293 cells were seeded at a density of 5.0 × 10^5^ cells/dish in 60-mm dishes. Transfection was performed with 1 μg DNA and 4 μl Lipofectamine 2000 in 150 μl OptiMEM medium (Invitrogen). One day after transfection, the cells were treated with 500 μg/ml G418 (Invitrogen) to select for the cells stably expressing the neomycin resistance gene and human IL-17RA. Where indicated, 293 cells were seeded into 60-mm cell culture dishes with approximately 0.8 × 10^6^ cells/dish. After 24 h incubation, the cells were treated with 20 mM LiCl (Sigma Aldrich), 10 μM MG132 (Sigma Aldrich), and/or 50 μg/ml cycloheximide (inhibitor of protein translation, Sigma Aldrich) for the indicated time periods. Then, the cell lysates were used for Western blot analysis.

### Mass spectrometry (MS) phosphopeptide mapping

293 cells were transiently transfected with pcDNA 3.1 expressing Flag-IL-17RA. 24 h later, Flag-IL-17RA protein was purified from whole cell lysate, using ANTI-FLAG^®^ M2 Affinity Gel (Sigma Aldrich) according to the manufacturer's instructions. The protein was separated on sodium dodecyl sulfate-polyacrylamide gel electrophoresis (SDS-PAGE) gel and stained with Coomassie Blue. The band corresponding to IL-17 RA band as detected by Western blot was excised from the gel and analyzed by MS. The gel pieces containing IL-17RA protein were dehydrated with acetonitrile for 10 min, vacuum dried, rehydrated with 5 mM Tris (2-carboxyethyl) phosphine hydrochloride in 25 mM ammonium bicarbonate (pH 8.5) at 37°C for 1 h, and subsequently alkylated with 20 mM iodoacetamide in 25 mM ammonium bicarbonate (pH 8.5) at room temperature for 1 h. The pieces were dehydrated with acetonitrile for 10 min, dried, and rehydrated with 35 μl of sequencing grade, modified trypsin (12.5 ng/μl) (Promega) in 25 mM ammonium bicarbonate (pH 8.5) at 37°C for overnight. Following the digestion, tryptic peptides were extracted three times with 50% acetonitrile containing 5% formic acid for 15 min each time with moderate sonication. The extracted solutions were pooled and evaporated to dryness under vacuum. To facilitate liquid chromatography (LC)-MS/MS characterization of phosphorylation sites, TiO2 based immobilized metal ion affinity chromatography (IMAC) was used to enrich the signals of phosphopeptides prior to MS. This enabled us to detect phosphopeptides with higher sensitivity [52]. Briefly, the peptides were dissolved in 20 μl of 5% formic acid plus 10% acetonitrile. The phosphopeptides were enriched using TiO2 TopTip (Glygen Inc.). The bound peptides were eluted with 0.5% NH_3_OH followed by acidification with 10% formic acid. Both flow-through and eluent were reconstituted in 50 μl 5% formic acid. LC-MS/MS analysis was performed on an integrated QSTAR XL nanoLC-MS/MS system (ABSCIEX) comprised of three micro-pumps with an autosampler, a stream select module configured for precolumn plus analytical capillary column, and a QSTAR XL mass spectrometer fitted with nano-sprayer III, operated under both Analyst 1.1 and MassLynx 4.0 control with a contact closure. Injected samples were first trapped and desalted isocratically on an Everest C18 precolumn (5 μm, 500 μm ID X 15 mm, Grace, Deerfield, IL) for 6 min with 0.1% formic acid delivered by the auxiliary pump at 40 μl/min, after which the peptides were eluted off from the precolumn and separated on an analytical C18 capillary column (15 cm × 100 μm i.D., packed with 3 μm Jupitor C18 particles, Phenomenex, Torrance, CA, USA) connected inline to the mass spectrometer, at 300 nl/min using a 50 min fast gradient of 5% to 60% acetonitrile in 0.1% formic acid and 0.0075% trifluoroacetic acid. MS/MS peak list was created from raw MS data using an Analyst “script” Mascot.dll. Then the peak list files were used to query human IL-17RA protein sequence using the MASCOT 2.4 from Matrix Science (London, UK) with the following parameters: peptide mass tolerance, 0.3 Da; MS/MS ion mass tolerance, 0.3 Da; allowed up to two missed cleavage; variable modifications considered were methionine oxidation, cysteine carboxyamidomethylation, phosphorylation (STY) with neutral losses of phosphoric acid and deamindation (N,Q). Only significant hits as defined by Mascot probability analysis were considered initially. Error tolerant search was used to assess the total sequence coverage of the protein. The phosphopeptides were identified based on neutral loss of 98 Da (corresponding to phosphoric acid) as a marker ion as well as at least 3 continuous ion series (y or b).

### Cell labeling with ^32^P orthophosphate

Approximately 2 million 293 cells were cultured in 100-mm cell culture dishes for overnight and transfected or co-transfected with 4 μg each of the indicated plasmids using the Lipofectamine 2000 according to the manufacturer's instructions. Two days after transfection, the cells were washed with phosphate-free DMEM and incubated in 5 ml phosphate-free DMEM medium containing 200 μCi ^32^P orthophosphate (MP Biomedical) for 4 h. The cells were washed with PBS twice and then lysed in 0.35 ml RIPA buffer (50 mM sodium fluoride, 0.5% Igepal CA-630 [NP-40], 10 mM sodium phosphate, 150 mM sodium chloride, 25 mM Tris pH 8.0, 1 mM phenylmethylsulfonyl fluoride, 2 mM ethylenediaminetetraacetic acid [EDTA], 1.2 mM sodium vanadate) supplementary with protease inhibitor cocktail (Sigma Aldrich). 0.3 ml of the extract was used for immunoprecipitation (IP) using ANTI-FLAG^®^ M2 Affinity Gel. Where indicated, an aliquot of IP products was treated with 20 units of calf intestine alkaline phosphatase (CIP, New England Biolab) at 37°C for 2 h. The samples were resolved by SDS-PAGE, transferred onto polyvinylidene difluoride (PVDF) membrane (BIO-RAD), and visualized by autoradiography followed by Western blot analysis.

### Western blot (or immunoblot) and dot blot analyses

Cell lysates or immunoprecipitates were subjected to 10% SDS-PAGE and transferred to PVDF membrane. For dot blot analysis, 1 μl peptide was printed onto nitrocellulose membrane (BIO-RAD) and air dried. The membranes were blocked with 5% nonfat dry milk in TBST buffer (25 mM Tris-HCl, 125 mM NaCl, and 0.1% Tween 20) for 1 h and probed with the indicated primary antibodies overnight. After washing 3 times with TBST buffer, the membranes were incubated with IRDye 800CW- or IRDye 680RD-conjugated secondary antibodies (LI-COR Biosciences) for 1 h. The results were visualized using an Odyssey Infrared Imager (LICOR Biosciences).

### *In vitro* kinase assay

For human peptides, 5 μg non-phosphorylated peptides were incubated with or without 1 μl (0.35 μg) of recombinant human GSK3α (Invitrogen) or 1 μl (500 units) recombinant human or rabbit GSK3β (R & D Systems or New England Biolab) in 9 μl GSK3 kinase buffer (New England Biolab) with or without 200 μM ATP (New England Biolab) at 30°C for 1 h. One μl sample was printed onto nitrocellulose membrane, air dried, and probed with anti-phospho-IL-17RA antibodies. For mouse peptides, 14 μg non-phosphorylated peptide was incubated with or without 2 μl (0.70 μg) of active recombinant GSK3α or 2 μl GSK3β (1000 units) in 18 μl GSK3 kinase buffer with 4 μCi γ-^32^P-ATP at 30°C for 1 h. About 20 μl samples were added to the filter (Microcon-10kDa Centrifugal Unit, Millipore) and centrifuged at 8000 × g for 30 min. This centrifugation was needed to remove ^32^P-labeled GSK3 due to auto-activation of the recombinant GSK3 during the kinase assay, as the molecular weight of GSK3 was larger than 10 kDa and hence could not pass through the 10 kDa-filter of Microcon-10kDa Centrifugal Units. Negative controls with GSK3 and ATP, but without the peptides, were included to rule out the possibility that any degraded/fragmented ^32^P-labeled GSK3 might be filtered through. Two μl of the filtrate (containing the molecules smaller than 10 kDa, including the peptides) were dot blotted onto a nitrocellulose membrane, air dried, and washed 3 times (10 minutes each) with 1% phosphoric acid solution to remove free γ-^32^P-ATP. The membrane was then analyzed by autoradiography. For full-length mouse IL-17RA, HA-tagged mouse IL-17RA [53] was first expressed in 293 cells and purified using IP. Aliquots of mouse IL-17RA were treated with recombinant human GSK3α or recombinant human or rabbit GSK3β as described above. The samples were analyzed using Western blot analysis with B4 antibody.

### Immunoprecipitation (IP)

293 cells were cultured in 100-mm cell culture dishes for overnight and transfected or co-transfected with 4 μg each of the indicated plasmids using Lipofectamine 2000 according to the manufacturer's instructions. Two days after transfection, proteins were extracted from the cells using RIPA lysis buffer. The extracts (0.3 ml each) were incubated with 15 μl ANTI-FLAG^®^ M2 Affinity Gel or 1 μg of the indicated primary antibody plus 20 μl of protein A sepharose CL-4B beads (GE Healthcare Bio-Sciences AB) overnight at 4°C with rotation. The samples were washed 3 times with phosphate-buffered saline (PBS) and boiled in 20 μl of SDS sample buffer for Western blot (WB) analysis, also called immunoblot (IB) analysis.

### Ubiquitination assays

Rabbit anti-K48-linkage specific polyubiquitin and anti-K63-linkage specific polyubiquitin monoclonal antibodies were purchased from Cell Signaling Technology. Mouse anti-HA antibodies were purchased from Covance. Plasmid pcDNA3.1-HA-Ubiquitin (HA-Ub, expressing wild-type ubiquitin) was provided by Dr. Hua Lu (Tulane University). Plasmids pRK5-HA-Ubiquitin-K48 (HA-K48, expressing ubiquitin with K48 only and other lysines were mutated to arginines) and pRK5-HA-Ubiquitin-K63 (HA-K63, expressing ubiquitin with K63 only and other lysines were mutated to arginines) were obtained from Addgene. Two million 293 cells were transfected or co-transfected with 4 μg each of the indicated plasmids using the Lipofectamine 2000 (Invitrogen) according to the manufacturer's instructions. Twenty-four h post-transfection, the cells were treated with or without MG132 for 6 h. Proteins were extracted from the cells and used for IP using ANTI-FLAG^®^ M2 Affinity Gel. The immunoprecipitates were subjected to Western blot analysis with the indicated antibodies.

### Quantitative real-time PCR (qRT-PCR) analysis

Human HeLa and 293-IL-17RA stable cells were treated with 2 μM AZD5363 (Selleck Chemicals, Inc.), 50 ng/ml insulin (Sigma Aldrich), and/or 20 ng/ml recombinant human IL-17A (R & D Systems) for 2 h. Mouse embryonic fibroblasts (MEFs) with wild-type *Gsk3β*^+/+^ or knockout *Gsk3β*^−/−^ were treated with 20 ng/ml recombinant mouse IL-17A, IL-17F, IL-17C, or IL-17E (R & D Systems) for 2 h. RNA was isolated for qRT-PCR analysis as described previously [33].

### Human tissue specimens

Human prostate and skin tissue samples were provided by Ochsner Clinic Foundation (for prostate tissues) and the Affiliated Hospital of Guangdong Medical College (for skin tissues) with approval of the Institutional Review Boards of Ochsner Clinic Foundation and Guangdong Medical College, respectively. A total of 56 prostate tissues (including prostate cancer with concurrent PIN lesions and para-tumor normal prostate tissues), 17 normal skin tissues, and 17 psoriasis skin tissues were used. These samples were archived and de-identified. They were fixed with 4% phosphate-buffered paraformaldehyde, embedded in paraffin blocks, and cut into 4-μm sections. The sections were de-paraffinized in xylene, hydrated using graded alcohols, and stained with hematoxylin and eosin (H & E) for histopathologic confirmation by two pathologists (W.W.T. and A.R.W.).

### Immunohistochemical (IHC) and immunofluorescence (IF) staining

IHC and IF double staining were conducted as described previously [17]. The antibodies used were rabbit anti-phospho-IL-17RA (B4) (1:500 dilution), rabbit anti-IL-17RA (1:100 dilution, H-168/sc-30175, Santa Cruz Biotechnology), mouse anti-Ki-67 (1:500 dilution, sc-23900, Santa Cruz Biotechnology), mouse anti-p63 (1:100 dilution, Biocare Medical), and Cy3–conjugated anti-mouse IgG and DyLight 488–conjugated anti-rabbit IgG (Jackson ImmunoResearch Laboratories). IHC double staining was conducted using a Vectastain^®^ ABC kit (Vector Laboratories) according to manufacturer's instructions.

### Animal models

Animal study was approved by the Animal Care and Use Committee of Tulane University. The breeding and genotyping protocols have been described previously, using *Pten*^loxp/loxp^ mice (strain C;129S4-*Pten*^tm1Hwu^/J) and PB-Cre4 mice (strain B6.Cg-Tg(Pbsn-cre)4Prb) that created *Pten* conditional knockout in mouse prostates with prostate cancer formation [17, 37]. Briefly, PB-Cre4 male mice were crossed to *Pten*^loxp/loxp^ female mice to produce *Pten*^loxp/WT^;Cre^+^ male breeders; then, *Pten*^loxp/WT^;Cre^+^ male mice were crossed to *Pten*^loxp/loxp^ female mice to produce *Pten*^loxp/loxp^;Cre^+^ male mice. At 3 weeks of age after genotyping and weaning, male mice with *Pten*^loxp/loxp^;Cre^+^ genetic background were randomized into a group (*n* = 10) fed with standard chow diet (13.2% calories by fat, cat# 5053, LabDiet, Brentwood, MO) or a group (*n* = 10) fed with high-fat diet (60% calories by fat, cat# D12492, Research Diets, New Brunswick, NJ). Animal body weight was measured weekly. At 30 weeks of age, animals were euthanized. Approximately 0.7 ml blood sample from each mouse was taken and transferred to a pre-coated EDTA vial (containing 0.4 mg ethylenediaminetetraacetic acid). The blood was immediately centrifuged and the plasma was stored at −80^°^C freezer until analysis. The plasma levels of IL-17A, insulin, IGF1, and leptin were measured using enzyme-linked immunosorbant assay (ELISA) kits (RayBiotech, Inc.) according to the manufacturer's instructions. Weights of whole animal, genitourinary (GU) block, inguinal fat, and epididymal fat were measured. Pathological examination of PIN and invasive prostate cancer, Western blot analysis of protein levels, and real-time quantitative PCR analysis of mRNA levels in the prostate tissues were performed as described previously [17].

### Statistical analysis

All *in vitro* experiments were replicated three times if not indicated otherwise. Statistical significance was determined by one-way analysis of variance (ANOVA) and Tukey's tests for multiple comparisons, or unpaired Student's *t* test for quantitative data with the assumption of normal distribution of data and equal sample variance, or by Kruskal–Wallis test for qualitative data. The tests were two-sided. A *P* value < 0.05 was considered statistically significant. Sample sizes were selected on the basis of preliminary results to ensure an adequate power. Animal number estimate was based on our previous studies [17, 18]. All cells and mice studied were included for statistical analysis. All data points were included. After genotyping, animals were randomized into either high fat-diet or standard chow-diet groups by flipping a coin. The examiners were blinded to the grouping of animals when performing pathological examinations.

## SUPPLEMENTARY MATERIALS FIGURES AND TABLE


