# Supplementary material for: Hyperinsulinemia enhances interleukin-17-induced inflammation to promote prostate cancer development in obese mice through inhibiting glycogen synthase kinase 3-mediated phosphorylation and degradation of interleukin-17 receptor
Source: Oncotarget. 2016 Feb 10;7(12):13651–66. doi: 10.18632/oncotarget.7296 (PMC4924668; doi:10.18632/oncotarget.7296)
Supplement: Supplementary file 1 [file oncotarget-07-13651-s001.pdf]

Hyperinsulinemia enhances interleukin-17-induced inflammation to promote prostate cancer development in obese mice through inhibiting glycogen synthase kinase 3-mediated phosphorylation and degradation of interleukin-17 receptor

Supplementary Materials

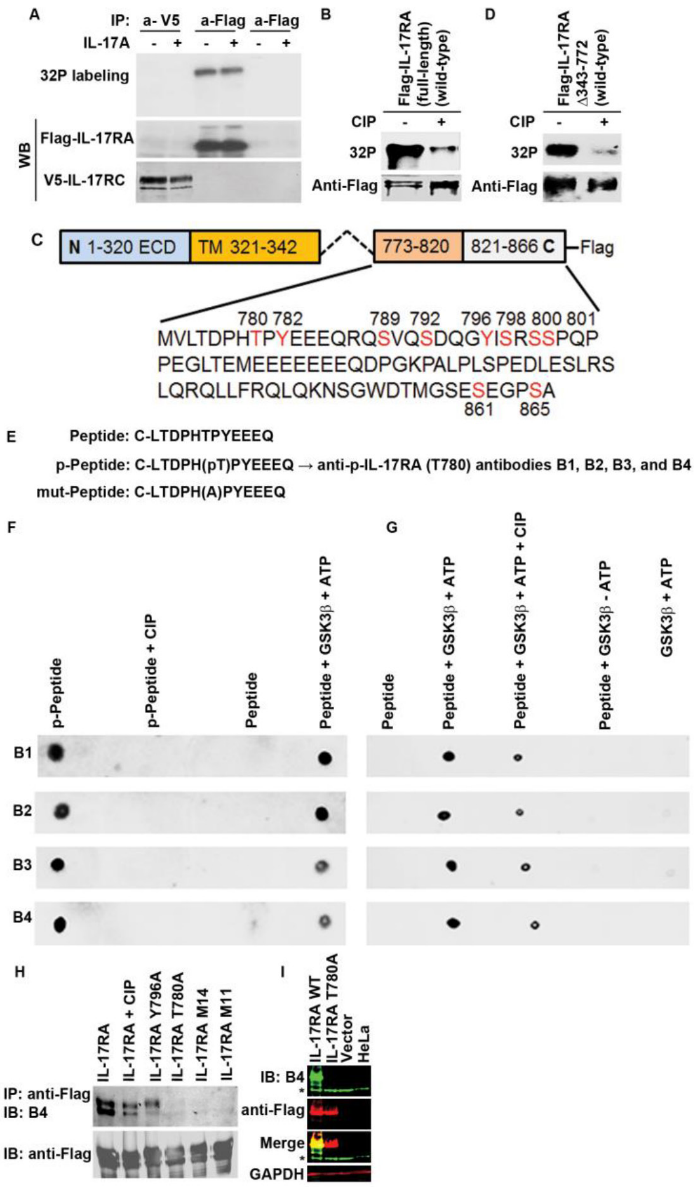

Supplementary Figure S1: IL-17RA is phosphorylated at multiple residues. (**A**) 293 cells were transiently transfected with V5-IL-17RC, Flag-IL-17RA, or empty vector; the cells were labeled with ^{32}P orthophosphate for 4 h and treated with or without IL-17A (20 ng/ml) for 20 min; cell lysates were used for IP with anti-V5 or anti-flag antibodies; after autoradiography (showing ^{32}P labeling), Western blot (WB) analysis was performed using anti-Flag and anti-V5 antibodies. (**B**) 293 cells were transiently transfected with wild-type full-length Flag-IL-17RA; the cells were labeled with ^{32}P orthophosphate for 4 h; cell lysates were used for IP with anti-flag antibodies; immunoprecipitated protein was split into two aliquots and treated with or without calf-intestinal alkaline phosphatase (CIP); after autoradiography (showing ^{32}P labeling), Western blot analysis was performed using anti-Flag antibodies. (**C**) Schematic illustration of the truncation construct of Δ343-772. The proximal 430 amino acids from the intracellular domain (343-772) were deleted; the remaining C-terminal domain contains 94 amino acids with 10 potential phosphorylation sites; this construct was used to make the constructs with all of the 10 potential phosphorylation sites mutated to alanine (Δ343-772 M10), with 9 potential

phosphorylation sites mutated to alanine while keeping T780 (Δ343-772 M9T780), or with 9 potential phosphorylation sites mutated to alanine while keeping Y796 (Δ343-772 M9Y796). (**D**) 293 cells were transiently transfected with wild-type Flag-IL-17RA Δ343-772 construct; the cells were labeled with ^{32}P orthophosphate for 4 h; cell lysates were used for IP with anti-flag antibodies; immunoprecipitated protein was split into two aliquots and treated with or without CIP; after autoradiography (showing ^{32}P labeling), Western blot analysis was performed using anti-Flag antibodies. (**E**) Sequences of non-phosphorylated peptide (Peptide), T780 phosphorylated peptide (p-Peptide), and T780A mutant peptide (mut-Peptide); each peptide was added a cysteine residue for conjugation procedure used in immunization of rabbits; p-Peptide was used for immunization of 4 rabbits, each producing anti-P-IL-17RA (T780) polyclonal antibodies named as B1, B2, B3, and B4; the Peptide was used to purify the antibodies and also used in *in-vitro* kinase assays with mut-Peptide as control. (**F**, **G**) Dot blot analysis using the antibodies to detect p-Peptide treated with or without CIP, and to detect Peptide treated with or without recombinant GSK3β, in the presence or absence of ATP. (**H**) 293 cells were transiently transfected with wild-type (WT) Flag-IL-17RA, Flag-IL-17RA with Y796A mutation, Flag-IL-17RA with T780A mutation, Flag-IL-17RA with mutation of 14 potential phosphorylation sites including T780A (IL-17RA M14), and Flag-IL-17RA with mutation of 11 potential phosphorylation sites including T780A (IL-17RA M11); IP was performed using anti-Flag antibodies; immunoprecipitated protein from the WT Flag-IL-17RA-transfected cells was split and treated with or without CIP; IB was performed using B4 and anti-Flag antibodies. The results showed that only IL-17RA with WT T780 was detected with B4 antibodies and P-IL-17RA level was reduced with CIP treatment. (**I**) 293 cells were transiently transfected with wild-type (WT) Flag-IL-17RA, Flag-IL-17RA with T780A mutation (IL-17RA T780A), or empty vector; HeLa cells were not transfected, in order to detected endogenous P-IL-17RA; whole cell lysates were used for IB using B4 and anti-Flag antibodies; GAPDH was detected for loading control; *indicates endogenous.

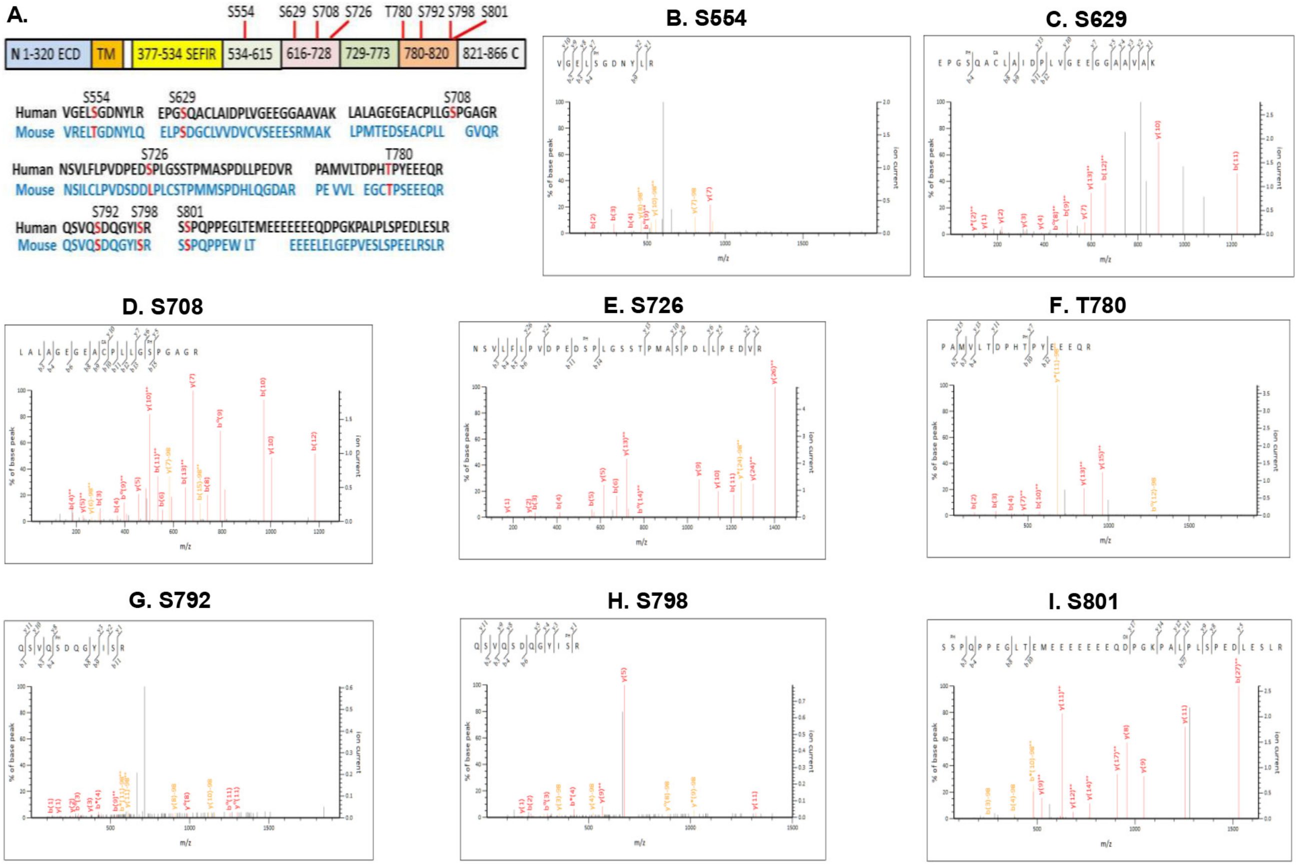

Supplementary Figure S2: Mass spectrometry phosphopeptide mapping of the phosphorylation sites of human IL-17RA. (**A**) Schematic illustration of human IL-17RA protein including the extracellular domain (ECD), transmembrane domain (TM), and C-terminal intracellular domain (containing the proximal similar expression to fibroblast growth factor genes, IL-17 receptors, and Toll-IL-1R domain; abbreviated as SEFIR domain); a summary of the 8 phosphorylation sites as identified by mass spectrometry phosphopeptide mapping is presented; a comparison of the human and mouse peptide sequences containing the phosphorylation sites is shown. (**B**–**I**) MS/MS spectrum obtained from the fragmentation of the precursor ions. Fragment ions corresponding to y-and b-ions were observed with the loss of 98 Da, attributable to the elimination of a neutral loss of phosphoric acid moiety; the phosphopeptides were identified based on neutral loss of 98 Da (corresponding to phosphoric acid) as a marker ion as well as at least 3 continuous ion series (y or b).

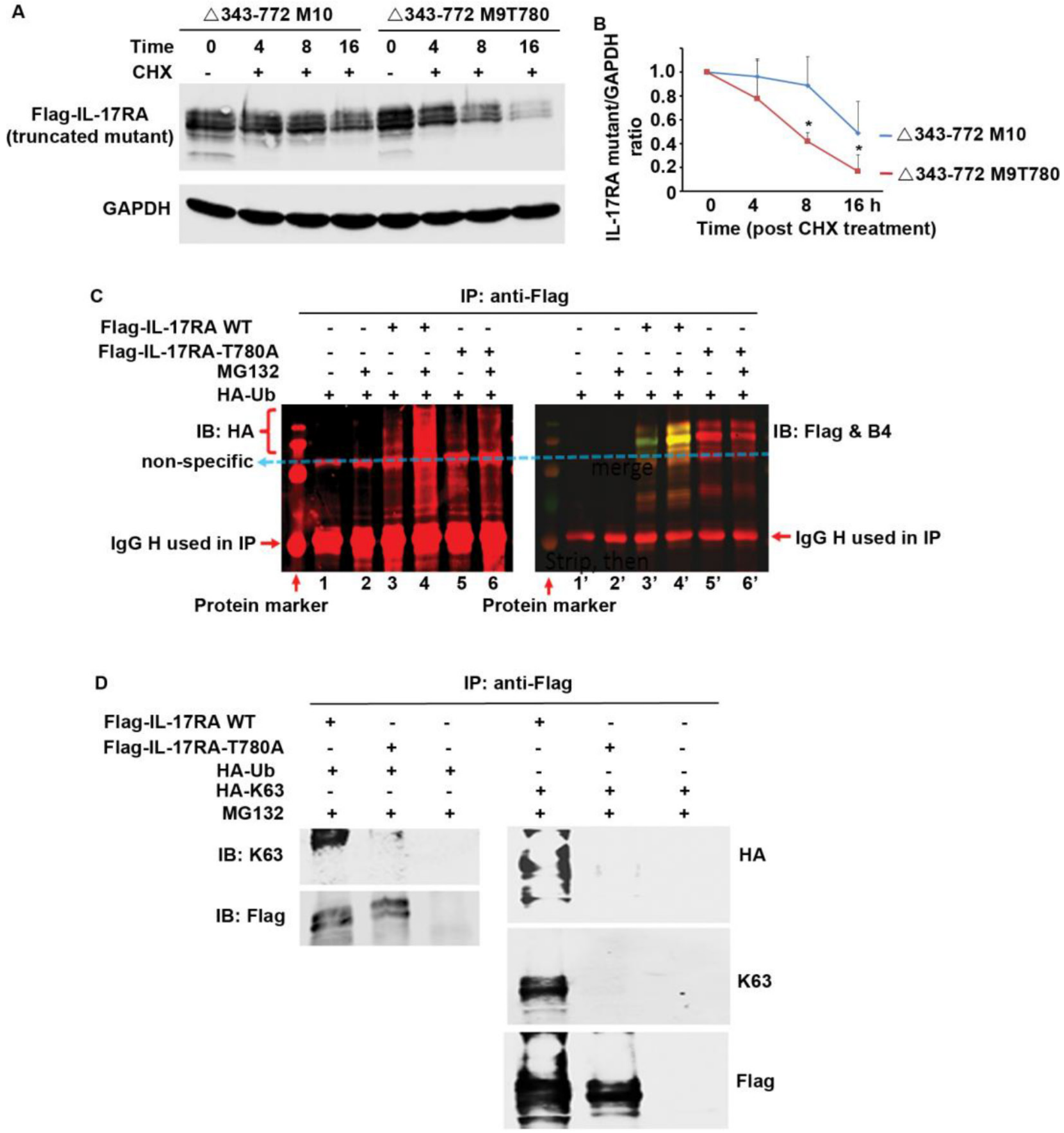

Supplementary Figure S3: Phosphorylation of IL-17RA at T780 leads to ubiquitination and degradation. (**A**) 293 cells stably expressing the truncated mutant constructs of Flag-IL-17RA were treated with 50 µg/ml cycloheximide (CHX, inhibitor of protein translation); Δ343-772 M10 construct has mutations in 10 potential phosphorylation sites including T780A; Δ343-772 M9T780 has mutations in 9 potential phosphorylation sites with a wild-type T780; whole cell lysates were used for Western blot analysis of Flag-IL-17RA and GAPDH. (**B**) Quantification of the Western blot signals using imaging analysis software installed in the Odyssey Infrared Imager; the signals of Flag-IL-17RA were normalized by the signals of GAPDH; the data represent mean ± standard deviation from 3 independent experiments ($n = 3$); $*P < 0.05$ compared to the Δ343-772 M10 construct. (**C**) Expanded figures of Figure 6E, showing that the non-specific band does not overlap with IL-17RA or IgG heavy chain; WT Flag-IL-17RA and Flag-IL-17RA-T780A mutant were co-transfected with HA-tagged ubiquitin (HA-Ub), with or without 10 µM MG132 treatment; IP was done using anti-Flag; IB was done using anti-HA, anti-Flag, and B4 antibodies, and then visualized with red or green fluorescent secondary antibodies. (**D**) Flag-IL-17RA and Flag-IL-17RA-T780A mutant were co-transfected with HA-tagged ubiquitin (HA-Ub) or K63-only ubiquitin (HA-K63, only K63 is available for polyubiquitination, while the other lysines are mutated to arginines), with 10 µM MG132 treatment; IP was done using anti-Flag; IB was done using anti-HA, anti-K63, or anti-Flag.

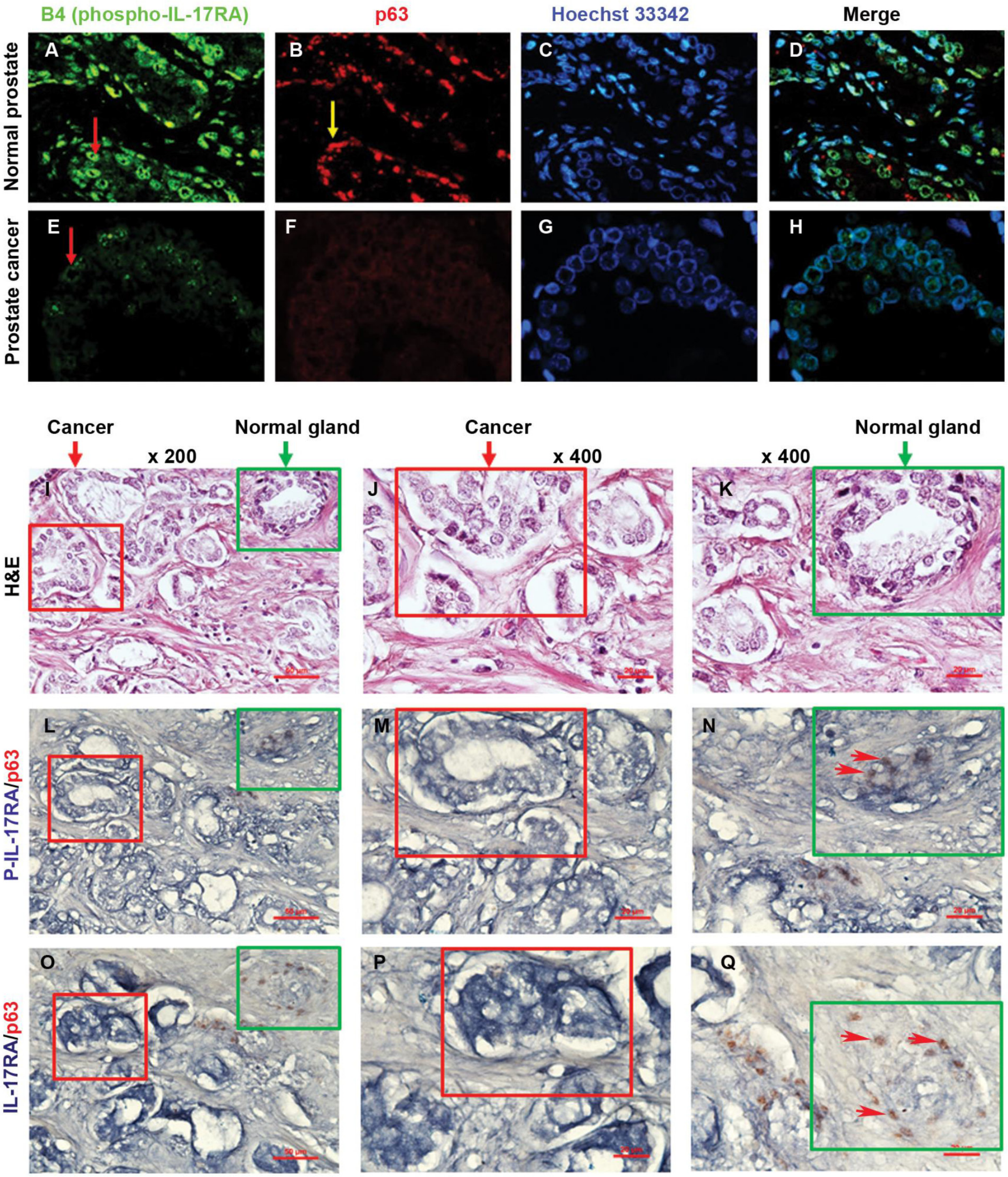

Supplementary Figure S4: Immunohistochemical staining of human prostate tissues. (**A**–**H**) Human normal prostate and prostate cancer tissues were triple stained with B4 (for P-IL-17RA, in green), p63 (for basal cells, in red), and Hoechst 33342 (for nuclei, in blue); arrows indicate the stained cells; magnification, x400. (**I**–**Q**) Human prostate tissues were double stained for phosphorylated IL-17RA (P-IL-17RA) using B4 (blue in color) or IL-17RA using anti-IL-17RA (H-168) (blue in color) and for basal cells using anti-p63 (red in color); representative regions of malignant glands (stained negative for p63) were highlighted in red windows while normal glands (stained positive for p63, indicated with arrowheads) were highlighted in green windows; scale bar, 50 µm (I,L,O) and 20 µm (J, K, M ,N, P, Q).

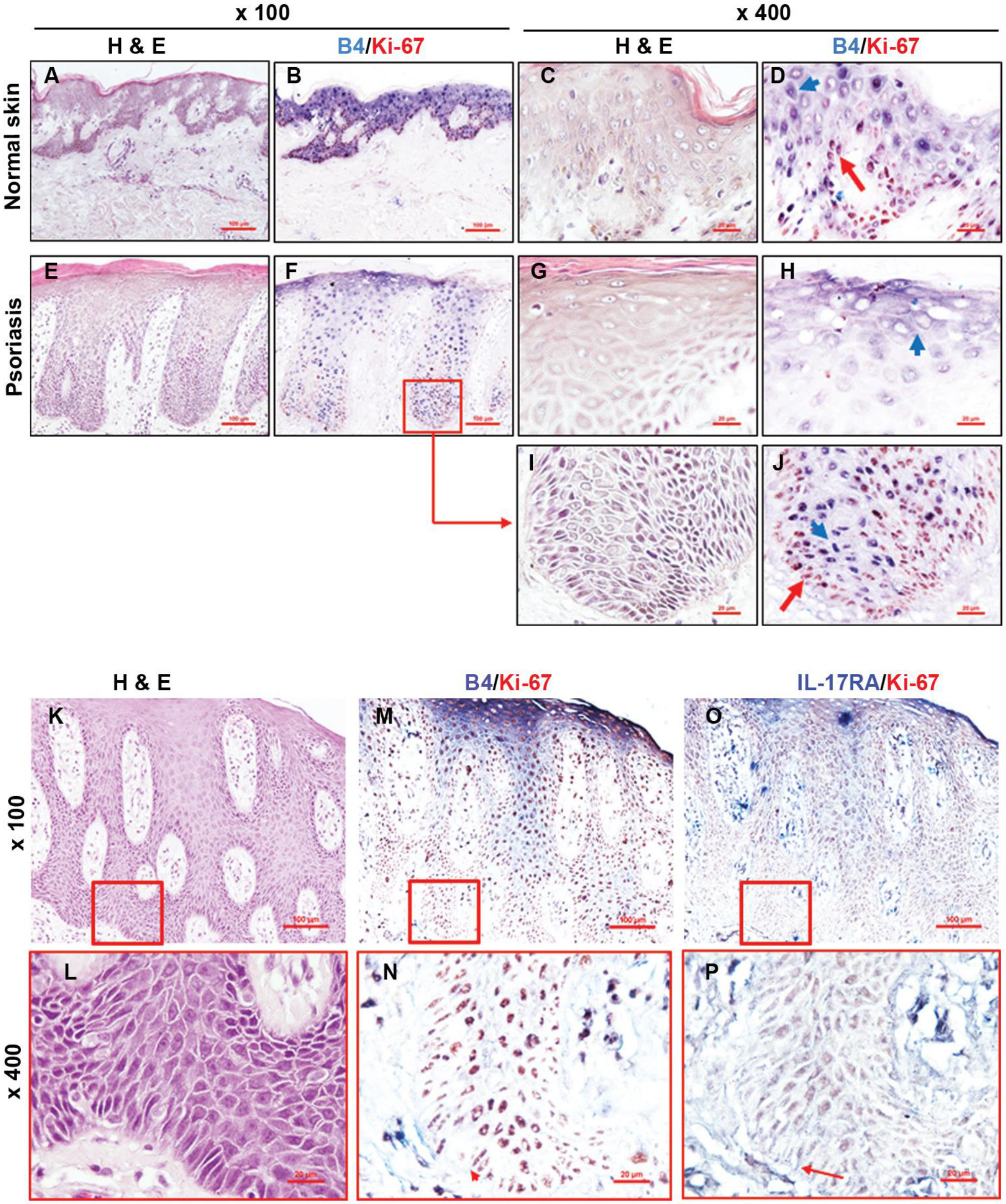

Supplementary Figure S5: Immunohistochemical staining of human skin tissues. (**A**–**J**) Human normal skin and psoriasis skin tissues were double stained for phosphorylated IL-17RA using B4 (blue in color, arrowheads) and for proliferating cells using anti-Ki-67 (red in color, arrows); scale bar, 100 µm (A, B, E, F) and 20 µm (C, D, G, H, I, J). (**K**–**P**) Human psoriasis skin tissues were stained with H & E, P-IL-17RA (using B4 antibodies, in blue color)/Ki-67 (in red color), or IL-17RA (in blue color)/Ki-67 (in red color) double staining, using Vectastain® ABC kit; arrowhead in (N) indicates the Ki-67-positive cells that were stained negative for P-IL-17RA; arrow in (P) indicate the Ki-67-positive cells that were stained positive for IL-17RA; scale bar, 100 µm (K, M, O) and 20 µm (L, N, P).

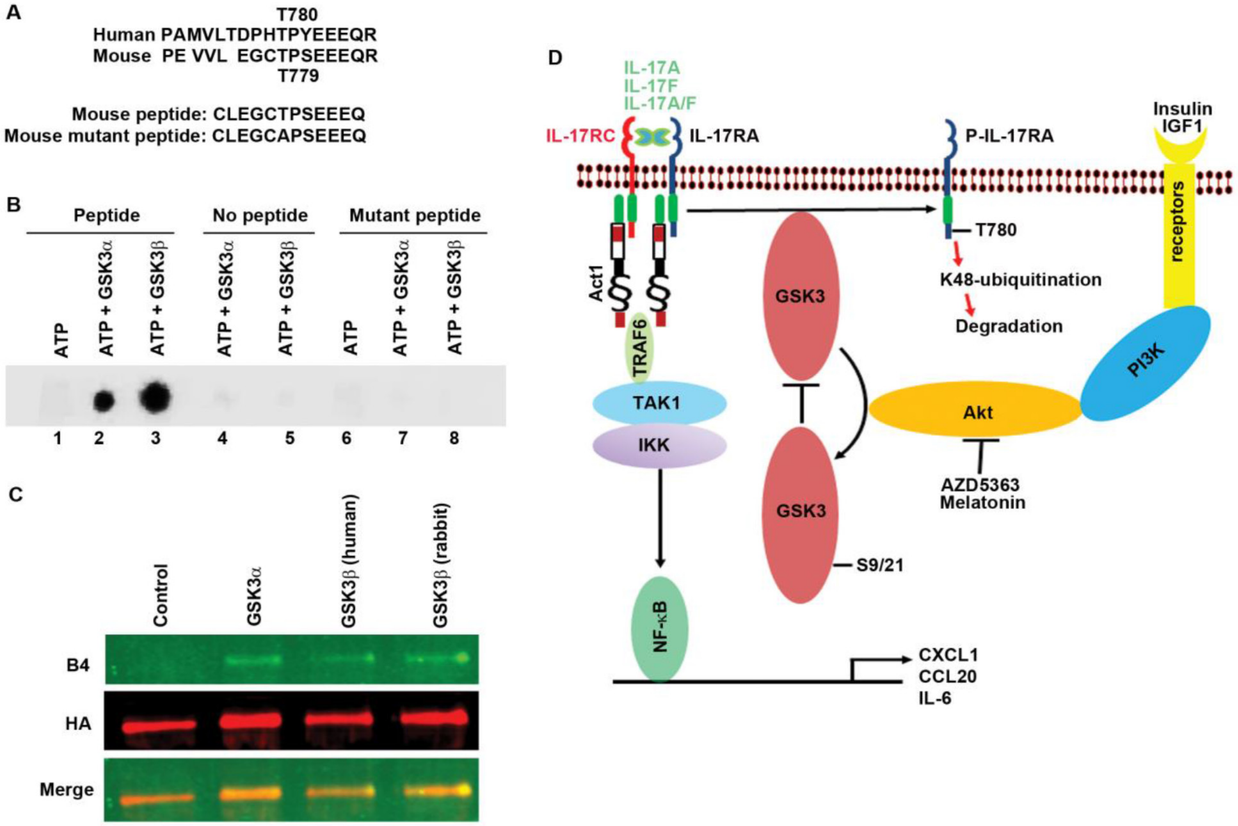

Supplementary Figure S6: Mouse IL-17RA peptide and full-length IL-17RA are phosphorylated by GSK3α and GSK3β. (**A**) Homology between human and mouse IL-17RA peptide sequences flanking human T780 and mouse T779, and wild-type and mutant mouse peptide sequences used in *in-vitro* kinase assays. (**B**) Mouse peptides were incubated with or without active recombinant GSK3α or GSK3β with γ-^{32}P-ATP. The products were centrifuged through Microcon-10kDa Centrifugal Unit (Millipore) to remove ^{32}P-labeled GSK3 due to auto-activation of the recombinant GSK3 during the kinase assay. The filtrates (containing the molecules smaller than 10 kDa, including the peptides) were dot blotted onto a nitrocellulose membrane, air dried, and analyzed by autoradiography. Groups treated with GSK3 and γ-^{32}P-ATP, but without the peptides, were included as negative control (lanes 4 and 5). Mouse mutant peptides were included as another negative control to confirm that T779 was the residue for phosphorylation (lanes 6–8). (**C**) HA-tagged mouse IL-17RA was first expressed in 293 cells and purified using IP with anti-HA. Aliquots of mouse HA-IL-17RA were treated with recombinant human GSK3α or recombinant human or rabbit GSK3β in *in-vitro* kinase assays. The samples were analyzed using Western blot analysis with B4 antibodies. The results showed very weak signals after GSK3 treatment, suggesting that mouse full-length IL-17RA was phosphorylated by GSK3 and that B4 antibodies (which were anti-human P-IL-17RA) were weakly cross-reacted with mouse P-IL-17RA. (**D**) Illustration of GSK3's role in the crosstalk between insulin/IGF1 and IL-17 signaling pathways. IL-17A, IL-17F, and IL-17A/F cytokines act through the IL-17RA/IL-17RC receptor complex to activate Act1-TRAF6-TAK1-IKK signaling cascade, thus activating NF-κB transcription factor for transcription of *CXCL1*, *CCL20*, and *IL-6*. GSK3 is constitutively active in phosphorylation of IL-17RA at T780, leading to K48-ubiquitination and proteasome-mediated degradation of P-IL-17RA. Consequently, IL-17RA level is reduced and IL-17 signaling is restricted by GSK3. In the presence of insulin and/or IGF1, phosphatidylinositol 3-kinase (PI3K)/Akt pathway is activated, resulting in phosphorylation of GSK3 at S9 (for GSK3β) or S21 (for GSK3α) and inhibition of GSK3 enzyme activities. Therefore, GSK3-mediated phosphorylation of IL-17RA is inhibited, resulting in increased levels of IL-17RA due to less degradation and consequently enhanced IL-17 signaling. Conversely, AZD5363 or melatonin can inhibit Akt, thus enhancing GSK3 activities to increase IL-17RA phosphorylation, ubiquitination, and degradation.

Supplementary Table S1: Potential phosphorylation sites of human IL-17RA

Extracellular and transmembrane domains			Intracellular domain		
**Site**	**Kinase**	**Score**	**Site**	**Kinase**	**Score**
S6	p38MAPK	0.52	T344	PKC	0.84
S6	GSK3	0.51	S356	CKII	0.61
S6	cdk5	0.60	S421	CKI	0.58
S32	PKA	0.70	S439	cdc2	0.53
S44	DNAPK	0.60	S441	cdc2	0.54
T51	PKC	0.77	T451	PKC	0.80
S55	PKA	0.65	T498	PKG	0.51
S61	cdc2	0.51	Y533	SRC	0.53
T69	p38MAPK	0.52	S554	CKII	0.52
T69	GSK3	0.50	S561	p38MAPK	0.55
T69	cdk5	0.68	S561	cdk5	0.57
S71	PKC	0.53	Y591	SRC	0.51
T85	DNAPK	0.56	S592	CKII	0.54
T85	ATM	0.50	S600	CKII	0.70
T102	PKC	0.57	S600	cdc2	0.52
S133	PKC	0.64	S629	DNAPK	0.65
T145	PKC	0.73	S708	p38MAPK	0.54
S147	PKC	0.55	S708	cdc2	0.55
Y157	SRC	0.51	S708	GSK3	0.51
T193	PKC	0.74	S708	cdk5	0.51
T194	p38MAPK	0.53	S715	PKA	0.77
T194	GSK3	0.51	S726	CKI	0.50
T194	cdk5	0.55	S726	GSK3	0.52
S198	CKI	0.53	S726	cdk5	0.57
S199	PKA	0.61	S731	cdc2	0.59
S201	DNAPK	0.51	T732	p38MAPK	0.50
T211	PKC	0.63	S736	p38MAPK	0.53
S220	PKA	0.63	S736	cdk5	0.54
S220	cdc2	0.54	S754	cdc2	0.50
T235	PKC	0.76	S761	cdc2	0.51
S244	CKII	0.59	T776	PKG	0.51
S264	PKA	0.67	T780	CKII	0.62
T269	PKC	0.83	T780	p38MAPK	0.54
S298	PKA	0.66	T780	GSK3	0.50
S302	CKII	0.55	Y782	EGFR	0.52
T309	p38MAPK	0.58	S789	PKC	0.51
T309	GSK3	0.53	S789	PKA	0.65
S329	PKA	0.64	S800	PKC	0.56
			S800	cdc2	0.53
			S801	cdk5	0.54
			T809	CKII	0.73
			S829	p38MAPK	0.54
			S829	GSK3	0.54

			S829	cdk5	0.56
			S852	PKA	0.67
			S852	cdc2	0.57
			T856	CKII	0.51
			S859	CKII	0.56
			S861	CKII	0.58

Human IL-17RA protein sequence (NP_055154.3) was analyzed with NetPhosK 1.0 Server (Technical University of Denmark, link: http://www.cbs.dtu.dk/services/NetPhosK/) using the default settings.
